# The prognostic value of CZT SPECT myocardial blood flow (MBF) quantification in patients with ischemia and no obstructive coronary artery disease (INOCA): a pilot study

**DOI:** 10.1007/s00259-023-06125-3

**Published:** 2023-02-14

**Authors:** Han Zhang, Federico Caobelli, Wenliang Che, Yan Huang, Yu Zhang, Xin Fan, Xueping Hu, Chong Xu, Mengyu Fei, Jiajia Zhang, Zhongwei Lv, Kuangyu Shi, Fei Yu

**Affiliations:** 1 Department of Nuclear Medicine, Shanghai Tenth People’s Hospital, Tongji University School of Medicine, Shanghai, 200072 China; 2 Institute of Nuclear Medicine, Tongji University School of Medicine, Shanghai, 200072 China; 3 Department of Nuclear Medicine, Inselspital, Bern University Hospital, University of Bern, Bern, Switzerland; 4 Department of Cardiology, Shanghai Tenth People’s Hospital, Tongji University School of Medicine, Shanghai, 200072 China; 5 Department of Radiology, Shanghai Tenth People’s Hospital, Tongji University School of Medicine, Shanghai, 200072 China; 6 Computer Aided Medical Procedures and Augmented Reality, Institute of Informatics I16, Technical University of Munich, Munich, Germany

**Keywords:** INOCA, CZT SPECT, Myocardial blood flow (MBF), Coronary flow reserve (CFR)

## Abstract

**Background:**

Despite the demonstrated adverse outcome, it is difficult to early identify the risks for patients with ischemia and no obstructive coronary artery disease (INOCA). We aimed to explore the prognostic potential of CZT SPECT in INOCA patients.

**Methods:**

The study population consisted of a retrospective cohort of 118 INOCA patients, all of whom underwent CZT SPECT imaging and invasive coronary angiography (ICA). Dynamic data were reconstructed, and MBF was quantified using net retention model. Major adverse cardiovascular events (MACEs) were defined as cardiovascular death, nonfatal myocardial infarction, nonfatal stroke, heart failure, late coronary revascularization, or hospitalization for unstable angina.

**Results:**

During a median follow-up of 15 months (interquartile range (IQR) 11–20), 19 (16.1%) MACEs occurred; both stress myocardial blood flow (sMBF) ($p<0.001$) and coronary flow reserve (CFR) ($p<0.001$) were significantly lower in the MACE group. Optimal thresholds of sMBF<3.16 and CFR<2.52 were extracted from the ROC curves, and both impaired sMBF (HR: 15.08; 95% CI 2.95–77.07; $p=0.001$) and CFR (HR: 6.51; 95% CI 1.43–29.65; $p=0.01$) were identified as prognostic factors for MACEs. Only sMBF<3.16 (HR: 11.20; 95% CI 2.04–61.41; $p=0.005$) remained a robust predictor when sMBF and CFR were integrated considered. Compared with CFR, sMBF provides better prognostic model discrimination and reclassification ability (C-index improvement = 0.06, $p=0.02$; net reclassification improvement (NRI) = 0.19; integrated discrimination improvement (IDI) = 0.10).

**Conclusion:**

The preliminary results demonstrated that quantitative analysis on CZT SPECT provides prognostic value for INOCA patients, which may allow the stratification for early prevention and intervention.

**Supplementary Information:**

The online version contains supplementary material available at 10.1007/s00259-023-06125-3.

## Introduction

Approximately 50%–70% of patients with chest pain and detectable myocardial ischemia do not have angiographic evidence of obstructive coronary artery disease (CAD) and are currently considered having ischemia and nonobstructive CAD (INOCA) [1]. Recent studies have shown that INOCA is a heterogeneous clinical condition with an increased risk of major adverse cardiac events (MACEs) and all-cause mortality compared to a normal population without ischemic heart disease [2, 3]. The development of INOCA is multifactorial, and coronary microvascular dysfunction (CMD) is considered a key factor in the development of adverse events [4]. CMD is defined as the alteration in the spectrum of epicardial, microvascular endothelial, or nonendothelial dysfunction [5, 6], leading to the decrease of myocardial blood flow (MBF) and coronary flow reserve (CFR) or myocardial flow reserve (MFR). However, the threshold and prognostic value of coronary flow quantification with different collection devices in INOCA patients remains unclear.

PET-CT with perfusion tracers like 13NH-ammonia and 82Rb-chloride has been extensively validated for the noninvasive assessment of CMD, allowing accurate calculation of MBF and CFR [7], but its widespread use is hampered by the need of an onsite cyclotron or costly generators for the production of positron-emitting perfusion tracers. In the latest years, attempts have been done to secure a role of new dedicated heart camera systems equipped with cadmium-zinc-telluride (CZT) solid-state detectors in the noninvasive assessment of MBF. In view of its advantages in spatial, temporal, and energy resolution over standard camera systems [8], an MBF quantitation is feasible and shows good consistency with PET-CT-based coronary flow values [9–12]. While the prognostic value of PET-derived MBF and CFR has been proved [13], still it needs to be demonstrated, whether such prognostic value in patients with INOCA also pertains to CZT SPECT imaging.

To fill this gap, we aim to investigate prognostic potential of CZT SPECT-derived MBF and CFR values in INOCA patients.

## Methods

### Patients

**Fig. 1:**
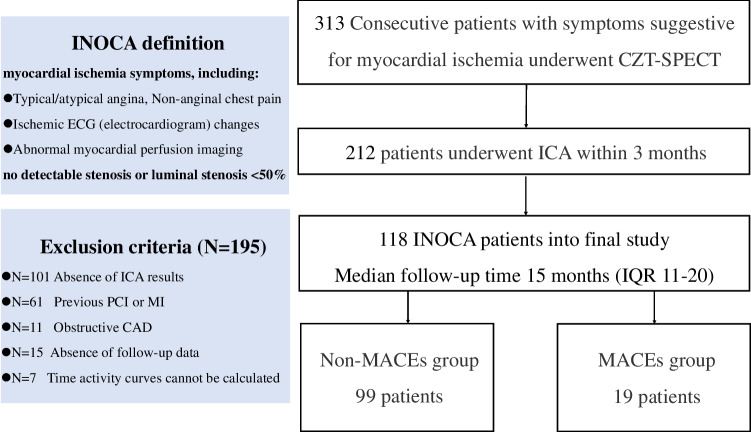
Patient flowchart

Three hundred thirteen consecutive patients with symptoms suggestive for myocardial ischemia were retrospectively evaluated. All patients were referred for CZT SPECT MBF quantification. Patients were excluded if invasive coronary angiography (ICA) results were not available ($n=101$), if a previous myocardial infarction (MI) or percutaneous coronary interventions (PCI) were reported ($n=61$), if they were already diagnosed with obstructive coronary artery disease ($n=11$), if follow-up data were not available ($n=15$), and, finally, if technical issues with CZT imaging prevented to outline time-activity curves ($n=7$). Other cardiac diseases that could induce anginal symptom (e.g., aortic stenosis or hypertrophic cardiomyopathy) were ruled out by cardiac ultrasound prior to the inclusion. After applying the exclusion criteria, 118 patients resulted eligible for the inclusion in our study (Fig. 1). ICA was performed within 3 months before CZT SPECT MBF quantification in 108 patients (91.5%) and within 1 month after in 10 patients (8.5%). In this cohort, INOCA was defined as the presence of symptoms suggestive for myocardial ischemia, including typical angina (meets the following three characteristics: constricting discomfort in the front of the chest or in the neck, jaw, shoulder, or arm; precipitated by physical exertion; and relieved by rest or nitrates within 5 min), atypical angina (meets two of these characteristics), or Non-anginal chest pain (meets only one or none of these characteristics) [14], which were associated with ischemic ECG (electrocardiogram) changes and/or abnormal myocardial perfusion imaging (MPI), without detectable stenosis or luminal stenosis <50% on ICA [1].

### Dynamic SPECT imaging protocol

All patients underwent one-day rest-stress myocardial perfusion imaging on a D-SPECT camera (Spectrum Dynamics Medical Ltd., Israel). Patients were instructed to discontinue β-blockers at least 48 h prior to the examination and discontinue any products containing methylxanthine including caffeinated coffee, tea, or other caffeine-containing medications at least 12 h prior to the examination and fasting for at least 3 h before the examination [15].

Rest imaging was always performed first. An initial test dose of approximately 37 MBq (1 mCi) of ^99m^Tc-sestamibi was injected to allow for a correct positioning of the patient’s heart within the field of view and to establish the scanning ROI. After this, patients were injected with a standard dose of 370 MBq (10 mCi) of ^99m^Tc-sestamibi, immediately followed by the acquisition of dynamic images in list mode over 6 minutes. 60–90 min later, standard static, rest perfusion images were acquired.

Thereafter, stress imaging was performed. All patients underwent a pharmacological stress test with adenosine (140 μg/(kg·min)). Three minutes after starting the infusion of adenosine, a standard stress dose of 925 MBq (25 mCi) of ^99m^Tc-sestamibi was injected, and the infusion of adenosine was continued for additional 3 minutes. Immediately after the injection of sestamibi, stress dynamic images were acquired over 6 min in list mode. Similar to rest imaging, 60–90 min later standard static stress perfusion images were acquired.

**Fig. 2:**
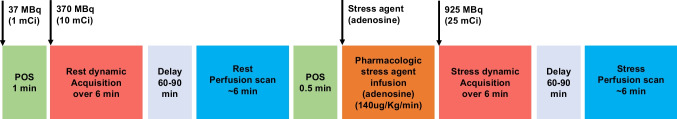
D-SPECT-CFR workflow

Dynamic imaging was reconstructed into 32 frames (21 frames 3s, 1 frame 9s, 1 frame 15s, 1 frame 21s, 1 frame 27s, and 7 frames 30s) as previously described [16]. An ordered subset expectation maximization (OSEM) iterative algorithm was used to reconstruct the images (4 iterations and 32 subsets). No attenuation correction or scatter correction was performed. The dynamic imaging protocol is illustrated in Fig. 2.

All dynamic data and corresponding perfusion information were analyzed using semiquantitative methods implemented in Corridor 4DM software (INVIA, Ann Arbor, MI, USA). Left ventricular (LV) endocardial and epicardial surfaces were automatically calculated after visual verification of the consistency of left ventricular contouring.

A midwall surface, determined equally distant between the endocardial and epicardial surfaces, is divided into 460 polar map sectors, where LV myocardial tissue time-activity curves (TAC) are the nearest neighbor sampled at the center of each sector across all time frames. The CFR analysis relies on region-of-interest (ROI) blood sampling by averaging a box-shaped region in the LV blood pool, specifically in the center of the LV on the short axis and centered at the basal valve plane along the long axis, across all time frames. The ROI size is two pixels wide on the short axis and 30 mm long on the long axis, to sample both the LV and left atrial cavities. The myocardial blood flow (MBF) was estimated for the global and regional myocardium through the previously established net retention model [17], and CFR was calculated as the ratio of the stress MBF to the rest MBF.

### Patients’ follow-up

Follow-up information was obtained through telephonic enquiries with each patient or their relatives; hospital history records collected by the referring cardiologist were also checked to retrieve relevant data [18, 19]. The median follow-up duration after CZT SPECT scan was 15 months (interquartile range (IQR) 11–20). The endpoint of major adverse cardiac events (MACEs) is defined as at least one of the following: cardiovascular death, nonfatal myocardial infarction, nonfatal stroke, heart failure, late coronary revascularization, or hospitalization for unstable angina [20].

### Statistical analysis

#### Subgroup analysis

Based on the follow-up results, the patients were divided into the MACEs group and the non-MACEs group. After verification of normal distribution with the Kolmogorov-Smirnov test, continuous data were presented as mean ± standard deviation (SD) or median ± IQR when not normally distributed, and categorical data were presented as frequency and percentage. The *χ*^2^ test was used for categorical data, and the independent samples t-test was used to compare the means of continuous data between the MACE group and the non-MACE group.

#### Prognostic predictors and construction of prognostic models

The cumulative incidence of MACEs was estimated using the Kaplan-Meier method and compared by the log-rank test. The time from the inclusion to the onset of a MACE was considered for this analysis. All variables were first assessed by univariate Cox proportional hazards regression analysis. Baseline variables that were considered clinically relevant or that showed a univariate relationship with outcome were entered into the multivariate Cox proportional hazards regression model [21]. Results were presented as hazard ratio (HR) and 95% confidence intervals (95% CIs). Receiver operating characteristic (ROC) curves and Youden’s index were used to calculate the best cutoff values of sMBF and CFR for the prediction of MACEs.

#### Comparison of increment prognostic value provided by sMBF and CFR

To assess the discrimination ability, we used C-index (area under the curve) for each predicted model at the median follow-up time (15 months (IQR 11–20)). To assess the incremental prognostic value of sMBF and CFR in the prognostic model, the net reclassification improvement (NRI) and integrated discrimination improvement (IDI) were calculated, in order to assess the difference in reclassification and discriminatory power.

A two-sided $p$ value of <0.05 was considered significant. All statistical analyses were performed using SPSS 16.0 for Windows (SPSS Inc., Chicago, IL, United States), MedCalc 18.0, and R language version 4.2.0 (Survival package, Nricens package, SurvivalNRIIDI package).

## Results

### Baseline clinical characteristics

This study included 118 INOCA patients (mean age 63.17±11.10 years, 45.8% male). The patients’ baseline characteristics in the respective groups are summarized in Table 1. All enrolled patients had symptoms suggestive for myocardial ischemia: atypical angina (52/118, 44.1%), typical angina (39/118, 33.0%), or nonanginal chest pain (27/118, 22.9%). Among them, 40/118 patients (33.9%) had ischemic ECG changes and 16/118 (13.5%) had abnormal MPI, defined as regional perfusion defects on visual analysis and/or wall motion abnormalities.

**Table 1:** Baseline characteristics of 118 INOCA patients

	Total ($n=118$)	Non-MACEs ($n=99$)	MACEs ($n=19$)	$p$ value
Patient characteristics				
Age (years)	$63.17\pm 11.10$	$62.25\pm 11.17$	$67.94\pm 9.62$	0.04
Male gender,$n \left(\%\right)$	54 (45.8%)	48 (48.5%)	6 (31.6%)	0.21
Height (cm)	$165.65\pm 8.14$	$166.10\pm 8.05$	$163.31\pm 8.41$	0.17
Weight (kg)	$68.23\pm 11.72$	$68.72\pm 11.74$	$65.68\pm 11.58$	0.30
Body mass index (kg/m^2^)	$24.78\pm 3.27$	$24.83\pm 3.36$	$24.50\pm 2.82$	0.68
Clinical symptoms				
Atypical angina,$n \left(\%\right)$	52 (44.1%)	43 (43.4%)	9 (47.4%)	0.75
Typical angina,$n \left(\%\right)$	39 (33.0%)	35 (35.3%)	4 (21.0%)	0.22
Non-anginal chest pain,$n \left(\%\right)$	27 (22.9%)	21 (21.2%)	6 (31.6%)	0.40
Risk factors				
Hypertension,$n \left(\%\right)$	68 (57.6%)	54 (54.5%)	14 (73.7%)	0.14
Diabetes,$n \left(\%\right)$	17 (14.4%)	13 (13.1%)	4 (21.1%)	0.47
Dyslipidemia,$n \left(\%\right)$	11 (9.3%)	10 (10.1%)	1 (5.3%)	1
Current smoker,$n \left(\%\right)$	24 (20.3%)	21 (21.2%)	3 (15.8%)	0.76
HDL (mmol/L)	$1.16\pm 0.28$	$1.16\pm 0.27$	$1.19\pm 0.30$	0.64
LDL (mmol/L)	$2.45\pm 0.84$	$2.54\pm 0.80$	$1.97\pm 0.92$	0.008
Cholesterol (mmol/L)	$4.10\pm 0.98$	$4.18\pm 0.97$	$3.65\pm 0.93$	0.03
Triglycerides (mmol/L)	$1.61\pm 0.87$	$1.65\pm 0.90$	$1.40\pm 0.69$	0.25
Glomerular filtration rate	$86.40\pm 17.94$	$87.91\pm 17.40$	$78.93\pm 19.20$	0.052
Baseline medications				
Aspirin,$n \left(\%\right)$	34 (28.8%)	30 (30.3%)	4 (21.1%)	0.58
Statins,$n \left(\%\right)$	82 (69.5%)	67 (67.7%)	15 (78.9%)	0.42
Beta-blockers,$n \left(\%\right)$	35 (29.7%)	27 (27.3%)	8 (42.1%)	0.27
CCB,$n \left(\%\right)$	45 (38.1%)	37 (37.4%)	8 (42.1%)	0.80
ACEI or ARB,$n \left(\%\right)$	38 (32.2%)	32 (32.3%)	6 (31.6%)	1
Nitrate,$n \left(\%\right)$	9 (7.6%)	7 (7.1%)	2 (10.5%)	0.64

MACEs occurred in 19 patients (16.1%), specifically hospitalization for unstable angina in 10 (8.5%), nonfatal myocardial infarction in 3 (2.5%), heart failure in 3 (2.5%), late coronary revascularization in 2 (1.7%), and nonfatal stroke in 1 (0.85%).

Patients were divided into two groups according to follow-up outcomes: MACEs and non-MACEs. Patients in the MACEs group were older (62.25±11.17 vs. 67.94±9.62, $p=0.04$) and had lower levels of LDL (2.54±0.80 mmol/L vs. 1.97±0.92 mmol/L, $p=0.008$) and total cholesterol (4.18±0.97 mmol/L vs. 3.65±0.93 mmol/L, $p=0.03$). All other baseline clinical variables, cardiovascular risk factors, and received medication were not different between groups.

### CZT SPECT MPI and MBF quantification results

**Fig. 3:**
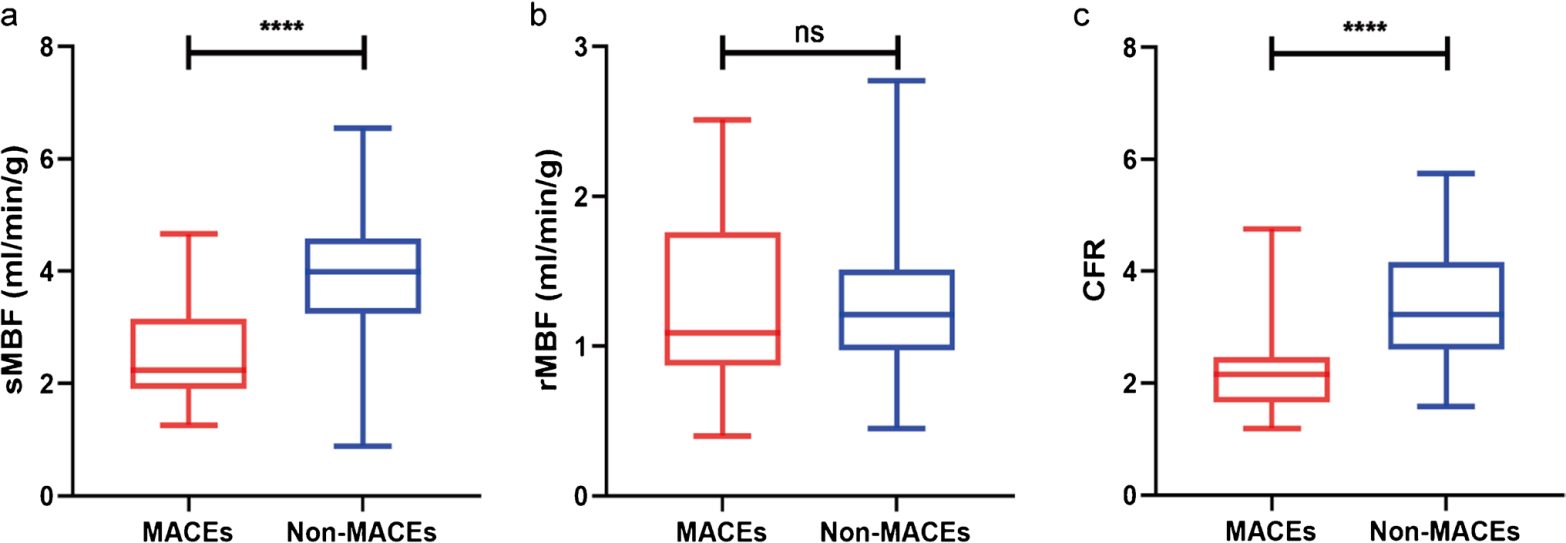
sMBF (**a**), rMBF (**b**), and CFR (**c**) levels between two groups. Differences were tested by independent t-test (ns=no significance, *p<0.05, **p<0.01, ***p <0.001)

An overview of MPI and CFR results for all patients is shown in Table 2. sMBF (2.53±0.86 vs. 3.96±1.01, $$p<0.001)$$ and CFR (2.28±0.93 vs. 3.41±1.01, $$p<0.001$$) were significantly lower in the MACEs group (Fig. 3). Similarly, partial LV functional parameters including stress LVEF, rest LVEF, SSS, stress TPD, and stress EXT were worse in the MACEs group (Table 2). Notably, rMBF was not statistically different between the two groups. Blood flow values correlated with patients’ age (Supplemental Figure 1).

**Table 2:** D-SPECT MPI and MBF quantification results of 118 INOCA patients

	Total ($n=118$)	Non-MACEs ($n=99$)	MACEs ($n=19$)	$p$ value
MPI findings				
Stress				
LVEF (%)	$67.25\pm 9.54$	$68.42\pm 9.34$	$61.16\pm 8.32$	<0.001
PER (−EDV/s)	$-3.69\pm 0.77$	$-3.71\pm 0.76$	$-3.61\pm 0.86$	0.61
PFR (EDV/s)	$2.51\pm 0.79$	$2.48\pm 0.75$	$2.68\pm 0.96$	0.30
SSS (IQR)	0 (0–15)	0 (0–7)	1 (0–15)	0.003
EXT (%)	0 (0–22)	0 (0–9)	1 (0–22)	0.024
TPD (%)	1 (1–20)	1 (0–8)	1 (0–20)	0.034
ESV (mL)	$22.46\pm 12.74$	$21.97\pm 12.66$	$24.47\pm 13.23$	0.45
EDV (mL)	$65.61\pm 20.99$	$65.88\pm 21.50$	$64.47\pm 19.26$	0.79
Rest				
LVEF (%)	$68.25\pm 10.34$	$69.97\pm 9.79$	$59.47\pm 8.82$	<0.001
PER (−EDV/s)	$-3.65\pm 0.82$	$-3.64\pm 0.75$	$-3.71\pm 1.12$	0.79
PFR (EDV/s)	$2.60\pm 0.77$	$2.55\pm 0.71$	$2.83\pm 1.04$	0.15
SRS (IQR)	0 (0–13)	0 (0–4)	0 (0–13)	0.07
EXT (%)	0 (0–20)	0 (0–5)	0 (0–20)	0.13
TPD (%)	0 (0–18)	0 (0–4)	0 (0–18)	0.06
ESV (mL)	$21.52\pm 13.55$	$20.82\pm 13.14$	$24.45\pm 15.15$	0.30
EDV (mL)	$61.69\pm 22.47$	$62.17\pm 23.54$	$59.74\pm 17.80$	0.67
SDS (IQR)	0 (0–7)	0 (0–7)	1 (0–5)	0.003
TID	$1.07\pm 0.13$	$1.07\pm 0.13$	$1.07\pm 0.10$	0.89
Stress MBF, mL/min/g	$3.73\pm 1.12$	$3.96\pm 1.01$	$2.53\pm 0.86$	<0.001
Rest MBF, mL/min/g	$1.24\pm 0.43$	$1.23\pm 0.40$	$1.26\pm 0.57$	0.87
CFR	$3.23\pm 1.08$	$3.41\pm 1.01$	$2.28\pm 0.93$	<0.001

### Prognostic value of CFR and sMBF

In our population, 6/16 patients (37.5%) with MPI perfusion/wall motion abnormalities experienced a MACE. Furthermore, a MACE was reported also in 15 out of 34 patients with reduced sMBF (44.1%) and in 15 out of 36 patients (41.7%) with impaired CFR. The characteristics of patients who experienced a MACE are reported in Supplemental Table 1, and Supplemental Table 2 displays differences between patients with hard events and those hospitalized for unstable angina.

**Fig. 4:**
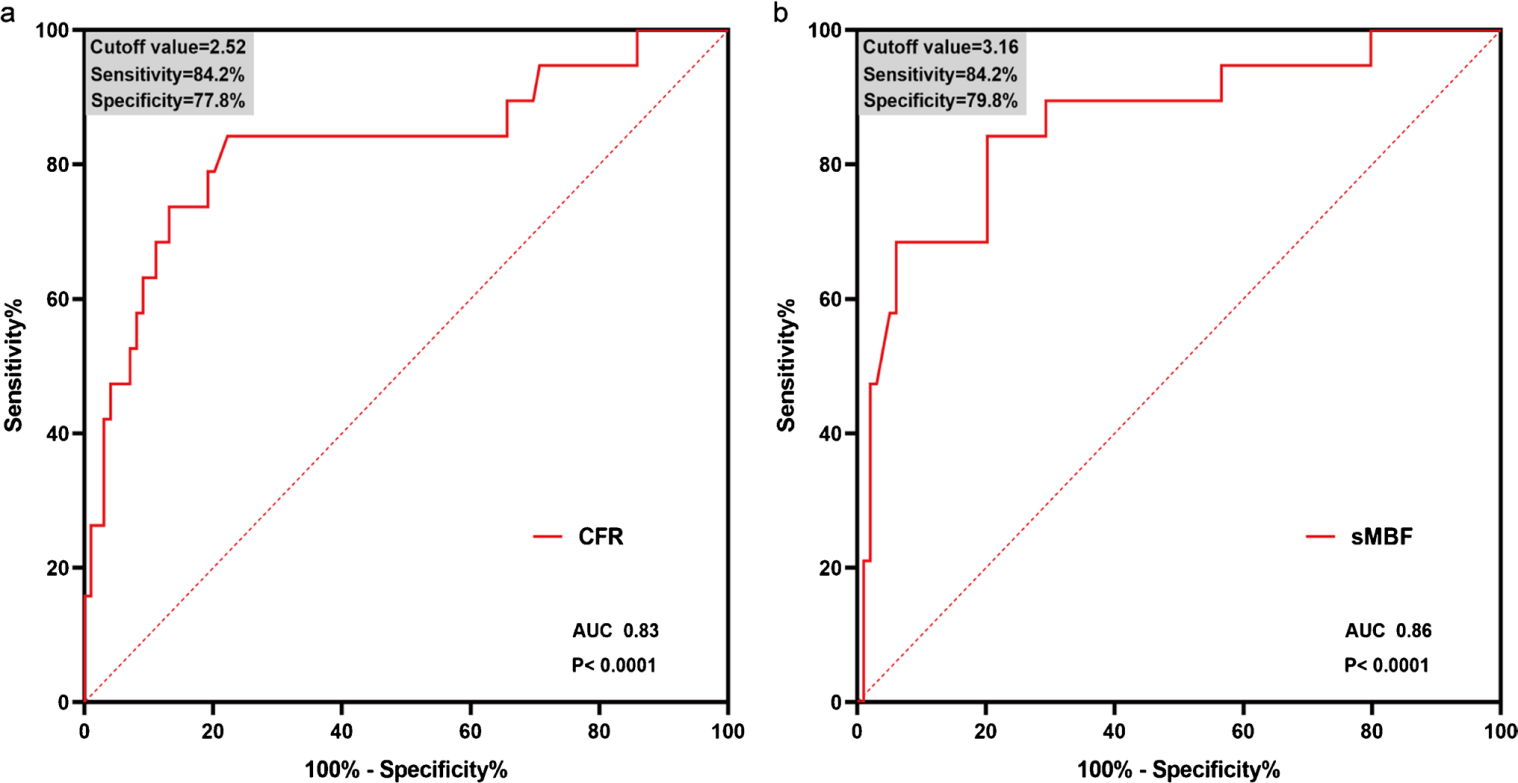
ROC curves of **a** CFR and **b** sMBF for predicting MACEs in INOCA patients

**Fig. 5:**
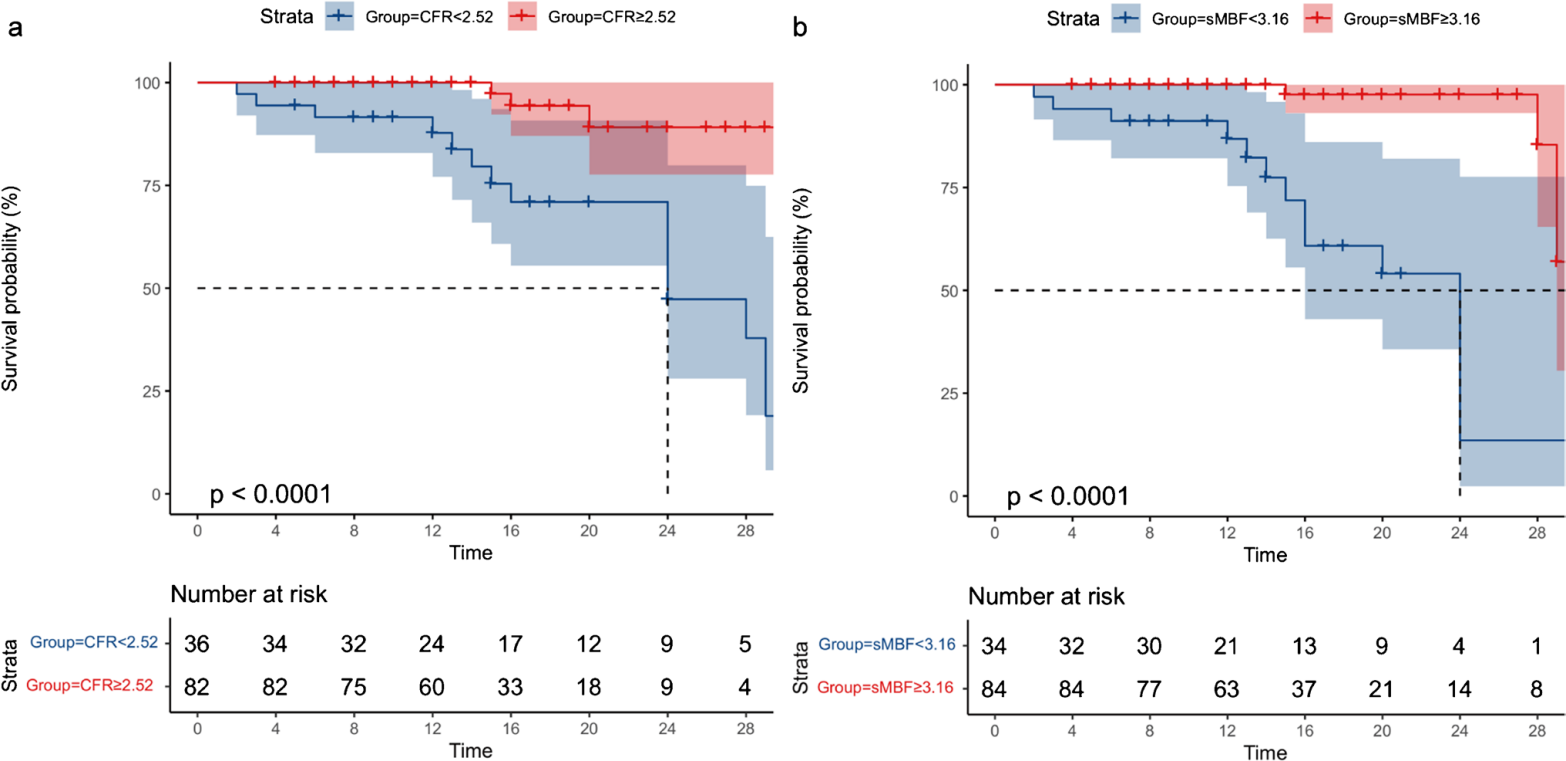
Kaplan-Meier curves in INOCA patients **a** strata by CFR<2.52 and **b** strata by sMBF<3.16 are shown

Based on the follow-up results, sMBF and CFR were independent predictors of the onset of MACEs ($p=0.001$ and $p=0.01$, respectively). Optimal thresholds for predicting MACEs were sMBF<3.16 (sensitivity 84.2%, specificity 79.8%, AUC 0.86, $p<0.0001$) and CFR<2.52 (sensitivity 84.2%, specificity 77.8%, AUC 0.83, $p<0.0001$) (Fig. 4). The Kaplan-Meier MACE-free survival analysis revealed a poor prognosis in patients with impaired sMBF (log-rank = 28.61, $p <0.0001$) and CFR (log-rank = 16.92, $p < 0.0001$) (Fig. 5).

The results of univariate and multivariable Cox regression analysis are reported in Tables 3 and 4. Since age and gender were considered clinically relevant with MACEs, we defined these two variables as possible clinical risk factors and included them into multivariate Cox regression. After adjusting for clinical risk factors and myocardial perfusion parameters, sMBF<3.16 (HR: 19.23; 95% CI 4.70–78.64; $p<0.001$; HR: 15.08; 95% CI 2.95–77.07; $p=0.001$) and CFR<2.52 (HR: 8.02; 95% CI 2.00–32.19, $p=0.003$; HR: 6.51; 95% CI 1.43–29.65; $p=0.01$) were still significant predictors of MACEs. Nevertheless, only sMBF<3.16 (HR: 11.20; 95% CI 2.04–61.41; $p=0.005$) still presented an independent predictor when CFR and sMBF were considered in combination.

**Table 3:** Univariable predictors of MACEs

Variables	Univariate hazard ratio (95% CI)	$p$ value
Age	1.03 (0.99–1.07)	0.11
Male	0.46 (0.17–1.22)	0.12
SSS	1.32 (1.16–1.50)	<0.0001
SRS	1.37 (1.17–1.60)	0.0001
SDS	1.45 (1.10–1.92)	0.009
CFR	0.48 (0.28–0.82)	0.007
sMBF	0.40 (0.25–0.63)	<0.0001
rMBF	0.98 (0.40–2.39)	0.97
LDL	0.64 (0.34–1.21)	0.17
Cholesterol	0.66 (0.39–1.12)	0.12
CFR<2.52	6.86 (1.94–24.22)	0.003
sMBF<3.16	12.42 (4.07–37.95)	<0.0001

**Table 4:** Multivariable predictors of MACEs

Variables	Adjust for CRF HR (95% CI)	$p$ value	Adjust for CRF, myocardial perfusion HR (95% CI)	$p$ value	Adjust for combined HR (95% CI)	$p$ value
CFR<2.52	8.02 (2.00–32.19)	0.003	6.51 (1.43–29.65)	0.01	3.83 (0.86–17.12)	0.08
sMBF<3.16	19.23 (4.70–78.64)	<0.001	15.08 (2.95–77.07)	0.001	11.20 (2.04–61.41)	0.005

*CRF* clinical risk factor, including age and gender; myocardial perfusion including SSS, SDS, and SRS

When sMBF and CFR were considered as continuous variables, the univariate analysis showed that both sMBF (HR: 0.40, 95% CI 0.25–0.63, $p<0.0001$) and CFR (HR: 0.48, 95% CI 0.28–0.82, $p=0.007$) were independent predictors (Table 3). In the multivariate regression analysis (Supplemental Table 3), after adjusting for clinical risk factors and myocardial perfusion parameters, sMBF (HR: 0.31; 95% CI 0.17–0.57; $p=0.0002$; HR: 0.35; 95% CI 0.17–0.69; $p=0.002$) and CFR (HR 0.39; 95% CI 0.21–0.70, $p=0.002$; HR: 0.45; 95% CI 0.25–0.81; $p=0.007$) were still significant predictors of MACEs. Similarly, sMBF (HR: 0.54; 95% CI 0.30–0.96; $p=0.03$) still presented an independent predictor when CFR and sMBF were considered in combination and without any categorization.

**Fig. 6:**
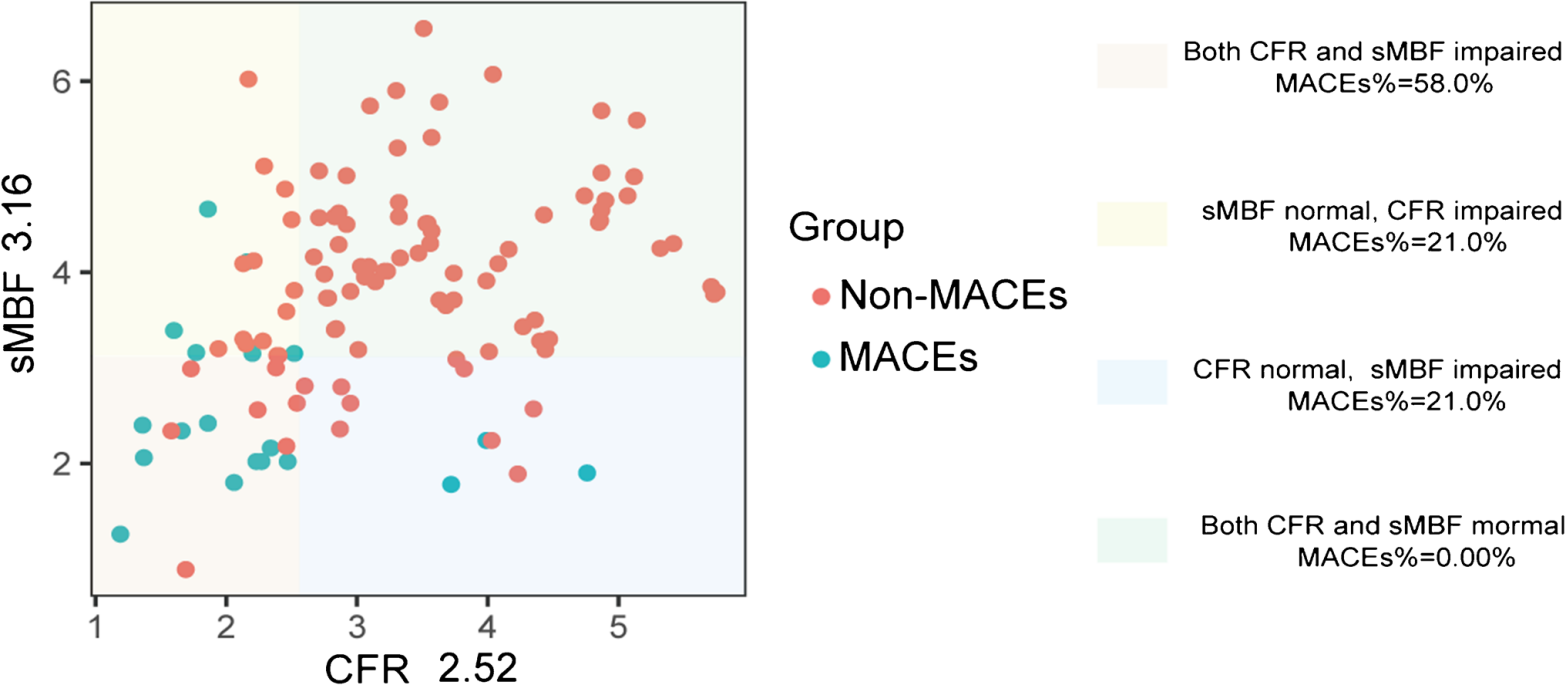
The scatter plot shows the consistency of CFR (<2.52) and sMBF (<3.16) impairment, with red dots representing non-MACE patients and blue dots representing MACE patients

**Fig. 7:**
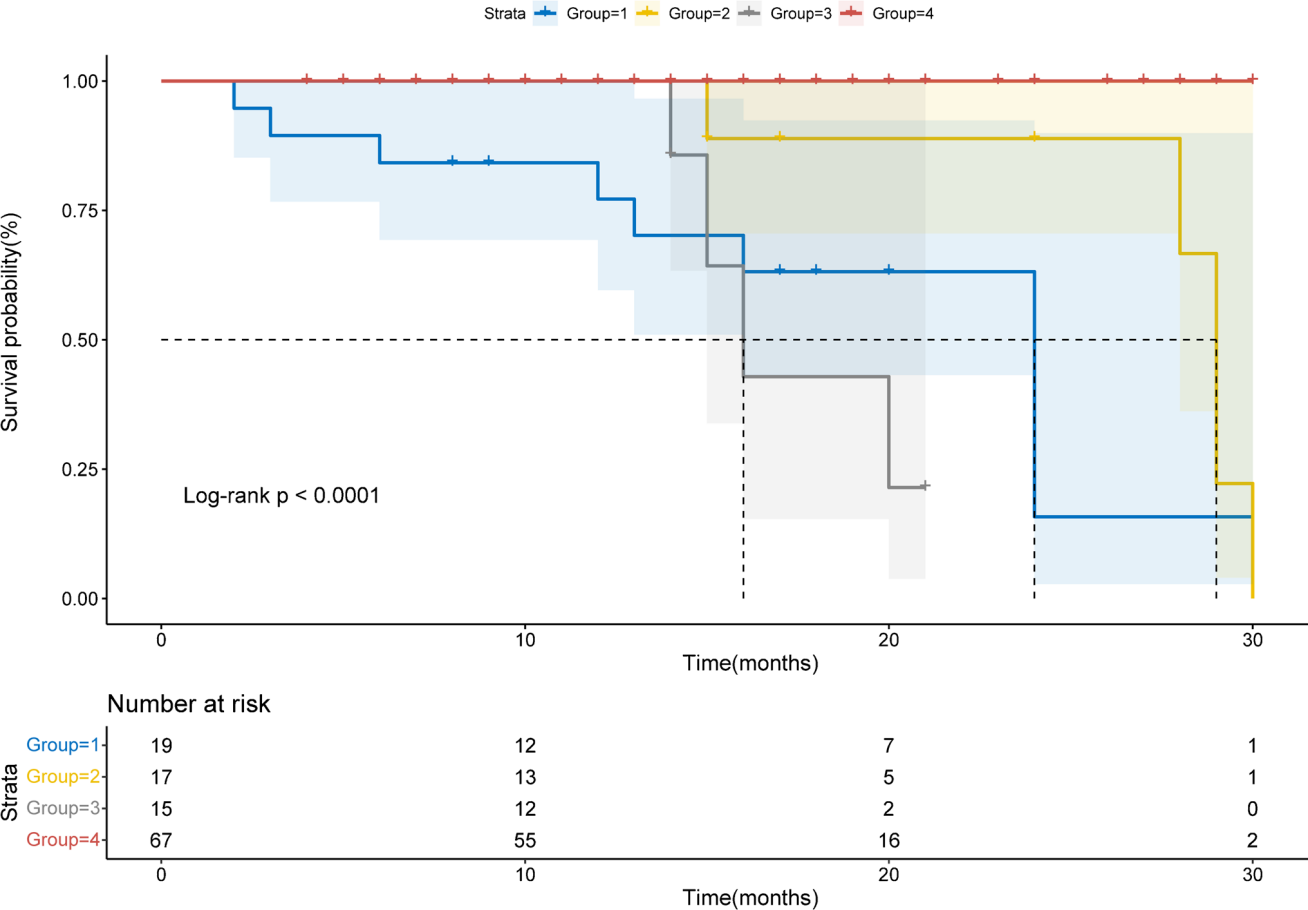
Kaplan-Meier curves are shown for 4 groups of INOCA patients subdivided by CFR and sMBF. Group 1 (CFR<2.52, sMBF<3.16); group 2 (CFR<2.52, sMBF≥3.16); group 3 (CFR≥2.52, sMBF<3.16); group 4 (CFR≥2.52, sMBF≥3.16)

**Fig. 8:**
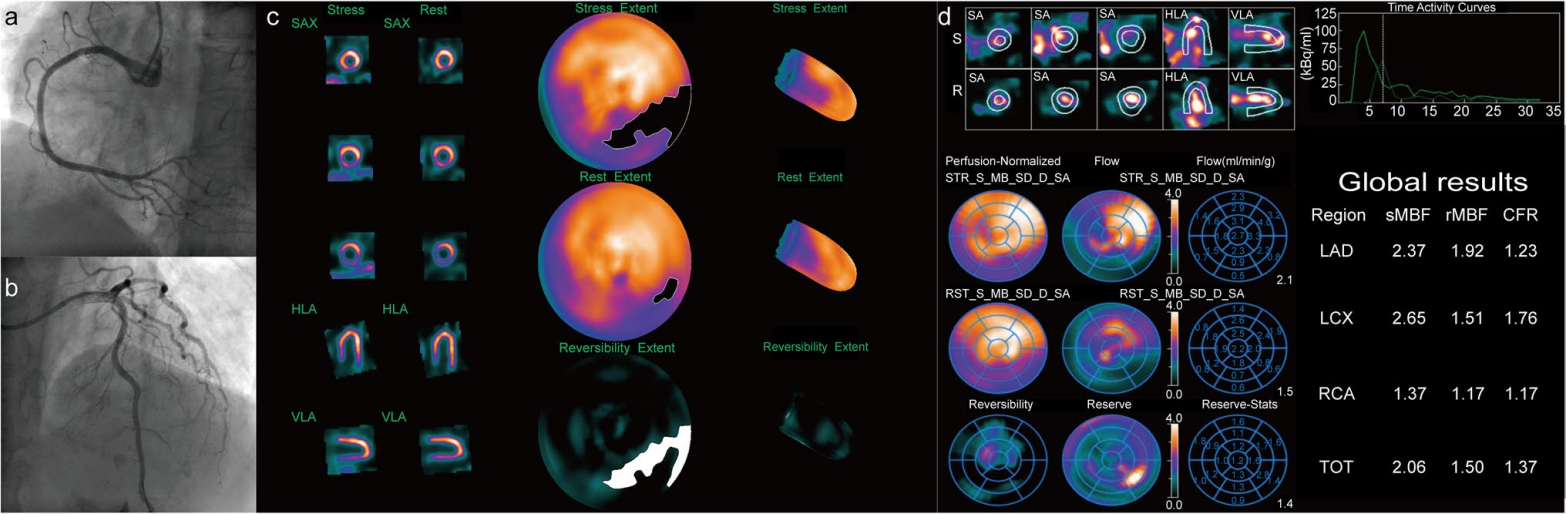
A 59-year-old male patient with hypertension and smoking history was referred for D-SPECT MBF quantification and ICA due to recurrent chest pain. **a** ICA indicates 30% stenosis of the right coronary artery (RCA), thrombolysis in myocardial infarction (TIMI) flow grade 3. **b** ICA indicates 30%–40% stenosis of the left anterior descending (LAD) coronary artery, TIMI flow grade 3. **c** Myocardial perfusion imaging (MPI) suggests partial reversible myocardial ischemia in the left circumflex artery (LCX) and RCA dominate segment. QPS/QGS quantitative analysis results: summed stress score (SSS) = 7, summed rest score (SRS) = 2, summed difference score (SDS) = 5. **d** MBF quantification showed significant decrease of MBF and CFR both regional and global. After 24 months of follow-up, this patient emerged with a nonfatal myocardial infarction

According to the consistency of CFR and sMBF, we further divided the patients into four groups: group 1 (CFR<2.52, sMBF<3.16, $n=19$, MACEs%=58.0%); group 2 (CFR<2.52, sMBF≥3.16, $n=17$, MACEs%=21.0%); group 3 (CFR≥2.52, sMBF<3.16, $n=15$, MACEs%=21.0%); group 4 (CFR≥2.52, sMBF≥3.16, $n=67$, MACEs%=0.00%). Scatter plot of the patient distribution is shown in Fig. 6. The survival analysis shows a similar risk of MACEs between groups 1 and 3 (log-rank = 0.075, $p=0.78$). Conversely, group 2 had lower risk of MACEs compared to group 1 (log-rank = 4.01, $p=0.04$). Furthermore, group 2 also had lower risk of MACEs compared to group 3 (log-rank = 5.31, $p=0.02$) (Fig. 7). One representative case is shown in Fig. 8.

Subsequently, we evaluated the performance of each prediction model by means of the C-index. Model 5 (C-index=0.92) and model 4 (C-index=0.92) showed the best discrimination ability, and the addition of CFR did not cause a statistical difference between the two models ($p=0.68$). sMBF (C-index=0.92) yielded significant incremental prognostic value over CFR (C-index=0.86; $p=0.022$). The predictive efficiency of all models with the addition of hemodynamic indicators was higher than that of clinical risk factors only and that of clinical risk factors combined with perfusion parameters ($p<0.05$ for all) (Table 5).

**Table 5:** Comparison of improved risk discrimination in five prognostic models

Model	C-index	$p$ value
Model 1: clinical risk factor (CRF)	0.62 (0.52–0.71)	Reference
Model 2: CRF + myocardial perfusion	0.80 (0.69–0.91)	<0.001
Model 3: CRF + myocardial perfusion + CFR	0.86 (0.79–0.93)	<0.001^a^, 0.057^b^
Model 4: CRF + myocardial perfusion + sMBF	0.92 (0.88–0.96)	<0.001^a^, 0.022^b^
Model 5: CRF + myocardial perfusion + CFR + sMBF	0.92 (0.87–0.96)	<0.001^a^, 0.68^b^

*CRF* clinical risk factor, including age and gender; myocardial perfusion, including SSS, SDS, and SRS

^a^Comparison with the reference model

^b^Comparison with the former model

Similar results were found when NRI and IDI were used to calculate the incremental prognostic value. The addition of CFR [NRI 0.45 (95% CI 0.03–0.72); IDI 0.28 (95% CI 0.12–0.54)] and sMBF [NRI 0.61 (95% CI 0.16–1.08); IDI 0.38 (95% CI 0.15–0.64)] to reference model 1 improves the reclassification and integrated discriminatory ability. Conversely, the addition of CFR to a model with clinical risk factor, MPI perfusion, and sMBF did not significantly increase [NRI 0.14 (95% CI −0.44–0.75); IDI −0.02 (95% CI −0.11–0.14)] (Table 6).

**Table 6:** Comparison of NRI and IDI in five prognostic models

Model	*NRI*^*a*^	*IDI*^*a*^	*NRI*^*b*^	*IDI*^*b*^
Model 1: clinical risk factor (CRF)	Reference	Reference	Reference	Reference
Model 2: CRF + myocardial perfusion	0.24 (−0.35–0.84)	0.27 (0.07–0.51)	0.24 (−0.35–0.84)	0.27 (0.07–0.51)
Model 3: CRF + myocardial perfusion + CFR	0.45 (0.03–0.72)	0.28 (0.12–0.54)	0.25 (−0.37–0.88)	0.015 (−0.11–0.23)
Model 4: CRF + myocardial perfusion + sMBF	0.61 (0.16–1.08)	0.38 (0.15–0.64)	0.19 (−0.77–1.07)	0.10 (−0.13–0.32)
Model 5: CRF + myocardial perfusion + CFR + sMBF	0.82 (0.20–1.11)	0.36 (0.17–0.66)	0.14 (−0.44–0.75)	−0.02 (−0.11–0.14)

*CRF* clinical risk factor, including age and gender; myocardial perfusion, including SSS, SDS, and SRS

^a^Comparison with the reference model

^b^Comparison with the former model

## Discussion

To the best of our knowledge, this is the first study exploring the prognostic value of CZT SPECT-derived sMBF and CFR in INOCA patients. Our study demonstrated that both sMBF and CFR are independent prognostic predictors of MACEs and provide incremental prognostic value over conventional MPI. Of note, the incremental value appears to be the highest if sMBF and CFR are simultaneously evaluated.

### CZT SPECT prognostic value in INOCA patients

Recently, a meta-analysis involving 35,039 INOCA patients showed that all-cause mortality and the rate of nonfatal myocardial infarction are higher for these patients than in the general population [22]. Hence, an accurate patients’ risk stratification and the early identification of risk factors for INOCA patients play a crucial role in clinical practice. MPI fits this need by providing information about coronary perfusion and left ventricular function, but standard MPI is hampered by suboptimal spatial and temporal resolution, which renders the quantification of MBF challenging on standard camera systems [23]. As such, information on microvascular impairment on standard MPI is limited. CZT SPECT is equipped with a more sensitive semiconductor cadmium zinc telluride (CZT) detector, compared to the conventional NaI-SPECT, provides 8–10 times increased sensitivity, 2 times increased spatial resolution [24, 25], which has been documented to provide better image quality and MPI prognostic information. In a study featuring an analysis of static perfusion imaging on CZT SPECT in 232 INOCA patients, Liu et al. showed that abnormalities on CZT SPECT-MPI are associated with adverse prognosis [26]. Similar results were reported by Mannarino et al. [27], wherein the analysis of regional perfusion abnormalities on CZT SPECT was predictive of MACEs in a large population of patients with suspected CAD. Furthermore, our previous study demonstrated that the left ventricular mechanical dyssynchrony (LVMD) evaluated by the CZT SPECT phase analysis technique allows for better risk stratification of INOCA patients [28].

As such, given the demonstrated prognostic value of different CZT-derived parameters, there is a clear rationale to secure a prognostic role for CZT SPECT-derived MBF, which represents a surrogate for the evaluation of microvascular dysfunction. In this regard, our paper confirmed the prognostic value of sMBF and CFR in INOCA patients, with a higher risk of developing MACEs if sMBF and CFR are out of certain reference range, and also demonstrated an incremental role over standard MPI indices. This aspect has an evident impact on clinical practice, wherein patients undergoing CZT SPECT MPI may be accurately risk-stratified, thus allowing for the choice of the most appropriate therapeutic regimen.

### Prognostic value of sMBF and CFR derived from CZT SPECT vs. PET

Since no epicardial coronary stenosis is present in INOCA patients, impaired MBF and CFR due to CMD have emerged as the third potential mechanism of myocardial ischemia in these patients [29]. In this regard, accurate measurements of MBF and CFR can further facilitate the risk stratification and thereby guide early treatment to reduce morbidity, mortality, and ultimately healthcare economic burden. Contemporary research has shown that PET-CT plays a pivotal role in the diagnosis of CMD and the prognostic assessment of INOCA patients [30]. Andrea et al. showed that after a median follow-up time of 8 years in 79 patients with chest pain and nonstenotic coronary arteries, both sMBF and CFR assessed by ^13^N-PET were predictors of MACEs [31]. Similar results were reported by Murthy et al. [32], wherein CFR as assessed by ^82^Rb-PET/CT was a robust prognostic factor regardless of gender in 1218 patients with no prior history and visual evidence of CAD.

While PET is still the most accurate molecular imaging methodology for the assessment of MBF, CZT SPECT has been gradually applied for flow measurement owing to higher availability of ^99m^Tc-labelled tracers and lower costs compared to PET. Several studies have now confirmed the good agreement in the calculation of MBF between CZT SPECT and PET-CT [9, 12]. It should be noted, however, that current studies on CZT SPECT-based MBF measurement still need more validation than those taking advantage of PET/CT in the setting of identification of obstructive CAD [10, 33]. Our study demonstrates that CZT SPECT can provide prognostic information similar to PET-CT, thus broadening the horizon for clinical application of CZT SPECT.

An important point is to establish thresholds in MBF able to predict the individual risk of cardiac disease progression. Previous studies featuring PET/CT MPI used CFR<2 as the cutoff value [34–36]. Several studies have calculated prognostic cutoff values for different cohorts. Bom et al. demonstrated global hMBF <2.65 mL/min/g and CFR <2.88 derived from [^15^O]H_2_O PET were optimal cutoff values for the prediction of MACEs in 648 patients with suspected or known CAD [37]. Similarly, Farhad et al. reported that sMBF of 1.8–2.6 mL/min/g and 1.8–2.4 for CFR allowed for a robust risk stratification [38].

Given the differences in hardware and radiopharmaceuticals, thresholds in CZT SPECT imaging need to be assessed. Our results show that threshold values of sMBF<3.16 and CFR<2.52 allow for good risk stratification of INOCA patients. It should be noted that thresholds of sMBF in our study are higher than those reported in previous PET studies, consistent with differences in scanners and in the extraction rate of SPECT tracers. The discrepancy is somewhat expected and consistent with the data from the literature. Acampa et al. showed in a head-to-head comparison between CZT SPECT and ^82^Rb PET that CZT-hyperemic MBF is systematically higher than that calculated on PET [11]. The same difference pertains to CZT SPECT compared to ^15^O-water PET/CT, as shown by Agostini et al. [12]. The contention may relate in the absence of correction for attenuation in CZT SPECT imaging, resulting in increased stress input curve, further causing the input curve to be out of calibration with consequent overestimated sMBF values [39, 40]. Furthermore, the differences in temporal resolution between CZT SPECT and PET and the different kinetic of the perfusion tracers, being the extraction rate of ^99m^Tc-labelled tracers much less proportional to coronary flow, may also explain the differences in calculated values between SPECT and PET.

It is still a matter of debate, which parameter (sMBF or CFR) is the best predictor of the onset of MACEs [41, 42]. According to the results of our study, sMBF yields more accurate prognostic information. This is consistent to what was reported from previous PET studies [35, 36] and may be explained by the fact that due to the absence of epicardial coronary stenosis, sMBF largely reflects the true coronary microvascular perfusion status of INOCA patients. Furthermore, impaired CFR is influenced by increased resting blood flow, which is not necessarily consistent with coronary stenosis, subsequently leading to less specific for predicting MACEs [37, 43]. Of note, also studies featuring a correction for heart rate and blood pressure in the calculation of rMBF showed better performance for sMBF compared to CFR. Recently, Bom et al. reported similar results in 648 suspected CAD patients with ^15^O-water PET; after adjustment for relevant risk factors and in combination with CFR, sMBF remained the only independent prognostic factor for death and MI [37]. Similarly, Farhad et al. demonstrated the additional prognostic value of ^82^Rb PET/CT-derived sMBF in 351 patients with known or suspected CAD [38]. Conversely, a recent study from Zampella et al. [44] suggested that impaired ^82^Rb PET/CT-based CFR may be a stronger predictor of cardiac events compared to sMBF in INOCA patients. This contention between their data and our study may relate firstly in the different imaging modality, but also in the different choice of endpoints. In fact, in our study, we included also hospitalization for unstable angina as cardiac event, and this choice caused a higher rate of MACEs (16.1%) compared to their population (7%). The rate of onset of MACEs in our population is conversely in line with data reported in studies featuring hospitalization for unstable angina as MACE [3]. Hence, the results cannot be completely compared but should rather be considered as complementary.

Moreover, while our study suggests that sMBF is superior to CFR, still the results show that the simultaneous evaluation of both sMBF and CFR yields improved prognostic value and should be therefore recommended, at least if values are assessed by means of CZT SPECT MPI.

A further clarification should be discussed about the impact of regional perfusion abnormalities on the prognosis of patients with INOCA. As a matter of fact, the results of the univariate analysis in our paper showed that higher perfusion scores are associated with a higher rate of MACEs. Moreover, some patients who experienced MACEs had perfusion abnormalities but preserved blood flows. Endothelial dysfunction may be considered as the main explanation, given the absence of detectable significant stenosis on ICA [45]. To note, although endothelial dysfunction alone may not be responsible for angina in some of these cases, when combined with another coronary abnormality of any degree, the probability for an additional effect would very likely increase [46]. It should also be noted that other factors involving cardiomyocytes (transcellular, intracellular, and mitochondrial) and the adventitia may contribute to the onset of ischemia. While these mechanisms should be kept on mind as possible additional predictors of a worse prognosis, the fact that the multivariate analysis in our work showed that sMBF after corrections remains the only independent predictor of MACEs should confirm its superiority over standard MPI in the prognostic assessment of patients with INOCA.

### Clinical implications

At present, PET-CT is not widely deployed even in high-income countries, and radiotracers and cost limitations still render SPECT-based MPI a more available diagnostic modality in patients with known or suspected CAD. As current study data have shown that the assessment of MBF and CFR with CZT SPECT using ^99m^Tc-trackers is feasible and reproducible [47], our study further expands on the clinical application of CZT SPECT in INOCA patients, by validating the prognostic value of MBF quantification and also confirming the superior prognostic value over standard, semiquantitatively assessed SPECT MPI. This latter aspect is expected to provide clinicians with new diagnostic strategies and prognostic prediction models, bearing importance in the choice of the most appropriate imaging modality for INOCA patients.

### Study limitation

This study has several limitations. First, FFR assessment was not available in our patients so that at least theoretically even lesions <50% may have a significant functional relevance. While this may have impacted the results in a small proportion of patients, still the vast majority of the patients had no detectable epicardial stenosis and therefore the impact of the lack of FFR in our cohort seems negligible. Second, as a proportion of INOCA patients recognizes symptoms due to coronary spasms, the implementation of an intracoronary acetylcholine test to assess endothelial function would have been useful to elucidate this potential mechanism. Unfortunately, the retrospective nature of the present study did not allow for this analysis. Finally, since this study was a single-center study and the limited number of patients may represent a limitation, a large population, multicenter external validation is needed to further verify the CZT SPECT-derived thresholds and prognostic values in different populations.

## Conclusion

The preliminary results demonstrated the role of quantitative CZT SPECT in the prognostic assessment of INOCA patients. sMBF and CFR were identified as predictors of adverse events, and the simultaneous evaluation of both yields the highest prognostic power. Our study contributes to an enhanced clinical translation of CZT SPECT, which should be considered as a robust and effective tool for prevention and early intervention of INOCA.

## Supplementary Information

Below is the link to the electronic supplementary material.

Supplementary file1 (DOCX 189 KB)

## Data Availability

The datasets generated during and/or analyzed during the current study are available from the corresponding author on reasonable request.
