# Supplementary material for: The prognostic value of CZT SPECT myocardial blood flow (MBF) quantification in patients with ischemia and no obstructive coronary artery disease (INOCA): a pilot study
Source: Eur J Nucl Med Mol Imaging. 2023 Feb 14;50(7):1940–53. doi: 10.1007/s00259-023-06125-3 (PMC10199834; doi:10.1007/s00259-023-06125-3)
Supplement: Supplementary file 1 — Supplementary file1 (DOCX 189 KB) [file 259_2023_6125_MOESM1_ESM.docx]

Supplemental Table 1. INOCA patients with MACEs

| Patients | Age  (years) | Gender | SSS | SRS | sMBF  (ml/min/g) | rMBF  (ml/min/g) | CFR | Type of events | Site of events | Timing  (months) |
| --- | --- | --- | --- | --- | --- | --- | --- | --- | --- | --- |
| No.1 | 65 | male | 8 | 6 | 2.16 | 0.92 | 2.34 | UA | / | 15 |
| No.2 | 86 | female | 1 | 0 | 1.9 | 0.40 | 4.76 | UA | / | 30 |
| No.3 | 69 | female | 3 | 0 | 2.34 | 1.41 | 1.66 | UA | / | 24 |
| No.4 | 69 | female | 5 | 2 | 3.16 | 1.79 | 1.77 | UA | / | 15 |
| No.5 | 59 | male | 7 | 2 | 2.06 | 1.50 | 1.37 | MI | Inferior | 24 |
| No.6 | 70 | male | 1 | 0 | 1.26 | 1.06 | 1.19 | CR | RCA | 29 |
| No.7 | 74 | female | 0 | 0 | 3.15 | 1.25 | 2.52 | HF | / | 14 |
| No.8 | 83 | female | 1 | 0 | 2.02 | 0.91 | 2.23 | HF | / | 24 |
| No.9 | 66 | female | 0 | 0 | 3.15 | 1.43 | 2.2 | UA | / | 13 |
| No.10 | 48 | female | 3 | 0 | 2.02 | 0.89 | 2.27 | Stroke | / | 2 |
| No.11 | 65 | male | 2 | 0 | 4.66 | 2.51 | 1.86 | MI | Inferior | 28 |
| No.12 | 55 | male | 0 | 0 | 3.39 | 2.12 | 1.6 | MI | Anterior | 30 |
| No.13 | 68 | female | 0 | 0 | 2.4 | 1.76 | 1.36 | UA | / | 16 |
| No.14 | 74 | female | 0 | 0 | 2.24 | 0.56 | 3.99 | UA | / | 20 |
| No.15 | 59 | female | 0 | 0 | 1.8 | 0.87 | 2.06 | UA | / | 6 |
| No.16 | 61 | female | 1 | 0 | 2.02 | 0.82 | 2.47 | UA | / | 3 |
| No.17 | 71 | female | 1 | 0 | 4.11 | 1.90 | 2.16 | HF | / | 29 |
| No.18 | 66 | male | 15 | 13 | 2.42 | 1.30 | 1.86 | CR | LAD, LCX and RCA | 12 |
| No.19 | 83 | female | 0 | 0 | 1.78 | 0.47 | 3.72 | UA | / | 16 |

SSS, summed stress score; SRS, summed rest score; SDS, summed difference score; sMBF, stress myocardial blood flow; rMBF, rest myocardial blood flow; CFR, coronary flow reserve; UA, hospitalization for unstable angina; MI, non-fatal myocardial infarction; CR, late coronary revascularization; HF, heart failure; Stroke, non-fatal stroke. LAD, left anterior descending artery; LCX, left circumflex artery; RCA, right coronary artery

Supplemental Table 2. Perfusion results between patients with hard events and hospitalization for unstable angina

|  | Hard Events  (n=9) | hospitalization for unstable angina  (n=10) | *P* value |
| --- | --- | --- | --- |
| perfusion findings |  |  |  |
| SSS (median ± IQR) | 1±4.5 | 0.5±3.5 | 0.008 |
| SRS (median ± IQR) | 0±1 | 0±0.5 | 0.14 |
| SDS (median ± IQR) | 1±2 | 0.5±2.25 | 0.02 |
| Stress TPD (%) | 2±4.5 | 1±3.5 | 0.01 |
| Rest TPD (%) | 0±0.5 | 0±0.25 | 0.14 |
| sMBF, ml/min/g | 2.79±1.11 | 2.30±0.5 | 0.25 |
| rMBF, ml/min/g | 1.49±0.57 | 1.04±0.52 | 0.09 |
| CFR | 1.89±0.44 | 2.63±1.13 | 0.08 |

SSS, summed stress score; SRS, summed rest score; SDS, summed difference score; TPD, total perfusion deficit; sMBF, stress myocardial blood flow; rMBF, rest myocardial blood flow; CFR, coronary flow reserve

Supplemental Table 3. Multivariable predictors of MACEs (CFR and sMBF as continuous variables)

| Variables | Adjust for CRF  HR (95% CI) | *P* value | Adjust for CRF, myocardial perfusion  HR (95% CI) | *P* value | Adjust for combined  HR (95% CI) | *P* value |
| --- | --- | --- | --- | --- | --- | --- |
| CFR | 0.39 (0.21-0.70) | 0.002 | 0.45 (0.25-0.81) | 0.007 | 0.66 (0.40-1.08) | 0.09 |
| sMBF | 0.31 (0.17-0.57) | 0.0002 | 0.35 (0.17-0.69) | 0.002 | 0.54 (0.30-0.96) | 0.03 |


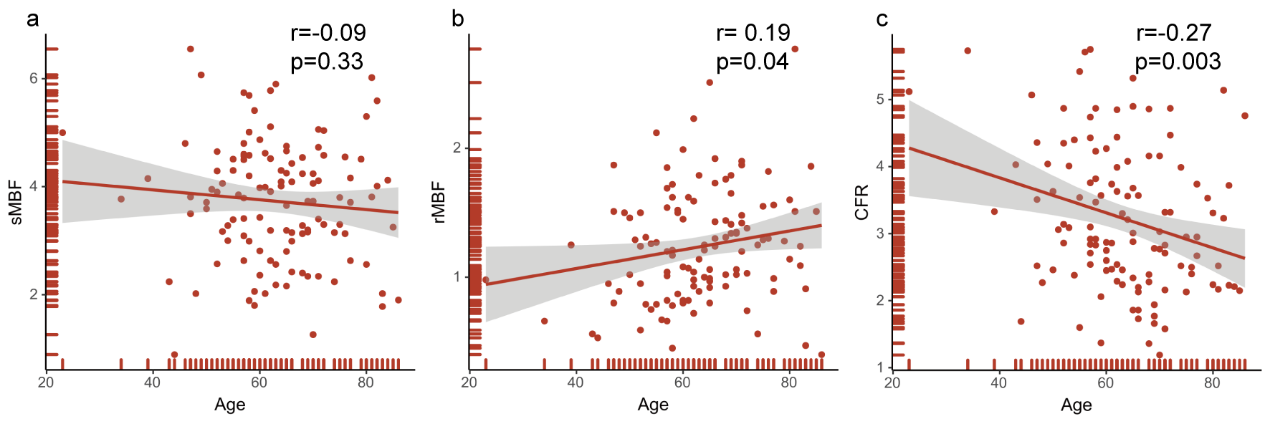


Supplemental **Fig 1 Correlation between age and sMBF (a), rMBF (b) and CFR (c)**
